# Nomogram based on systemic inflammatory response markers predicting the survival of patients with resectable gastric cancer after D2 gastrectomy

**DOI:** 10.18632/oncotarget.8788

**Published:** 2016-04-18

**Authors:** Jianjun Liu, Qirong Geng, Shangxiang Chen, Xuechao Liu, Pengfei Kong, Zhiwei Zhou, Youqing Zhan, Dazhi Xu

**Affiliations:** ^1^ State Key Laboratory of Oncology in South China, Collaborative Innovation Center for Cancer Medicine, Guangzhou, China; ^2^ Department of Gastric and Pancreatic Surgery, Sun Yat-sen University Cancer Center, Guangzhou, China; ^3^ Department of Hematology Oncology, Sun Yat-sen University Cancer Center, Guangzhou, China

**Keywords:** resectable gastric cancer, nomogram, survival analysis, systemic inflammatory response

## Abstract

This study aimed to construct a nomogram to predict survival of patients with resectable gastric cancer (RGC) based on both clinicopathology characteristics and systemic inflammatory response markers (SIRMs). Of 3,452 RGC patients after D2 gastrectomy at the Sun Yat-sen University Cancer Center, 1058 patients who met the inclusion criterion were analyzed. The patients operated on from January 1, 2005 to December 31, 2009 were assigned to the training set (817 patients) to establish a nomogram, and the rest (241 patients) were selected as validation set. Based on the training set, seven independent risk factors were selected in the nomogram. The calibration curves for probability of 1-year, 3-year and 5-year overall survival (OS) showed satisfactory accordance between nomogram prediction and actual observation. When the metastatic lymph node stage (mLNS) is replaced by metastasis lymph node ratio (mLNR) in validation set, the C-index in predicting OS rise from 0.77 to 0.79, higher than that of 7th American Joint Committee on Cancer 7th (AJCC) staging system (0.70; p<0.001). In conclusions, the proposed nomogram which including mLNR and routine detected SIRMs resulted in optimal survival prediction for RGC patients after D2 gastrectomy.

## INTRODUCTION

Gastric cancer is the second leading cause of cancer-related death worldwide, and the 5-year survival is 28% in 2014 [1]. Although the incidence of gastric cancer has declined in the past several years, it remains the most common cancer in many geographic regions, including Eastern Asia, Eastern Europe and Southern/Central American [2].

Currently, the widely used AJCC 7th TNM staging system is the most common tool to predict survival for gastric cancer. It stratifies RGC patients into seven groups [3]. Additionally, our previous study showed the ratio between metastatic and examined lymph nodes (mLNR) based staging system (TRM) is superior to the AJCC 7th system. The new classification can reduce stage migration and provide more reliable prognostic information [4–6].

Recently, nomograms have been established in many types of cancers [7–12]. It is a simple tool to predict individualized survival by incorporating more risk factors such as sex, age and size of the tumor [13]. However, besides tumor-related factors, the survival of cancer patients is also dependent on host's reaction to the tumor. Especially the host inflammatory response markers (SIRMs), a powerful prognostic factors, has not ever been included as a risk group in previous nomograms [14–24].

In this study, we established a nomogram combined clinicopathology characteristics and routine detected SIRMs, to determine whether this model predict a more accurate survival for RGC patients following D2 resection when compared with the currently TNM staging system.

## RESULTS

### Clinical characteristics of patients

A retrospective study was conducted on a primary cohort, who underwent D2 resection between January 1, 2005 and December 31, 2010 at the Sun Yat-sen University Cancer Center. Of 3,452 RGC patients, 1058 patients met all the inclusion and exclusion criteria. The patients operated between January 1, 2005 and December 31, 2009 were assigned to the training set (817 patients) for the construction of a nomogram, and the rest (241 patients) between January 1, 2010 and December 31, 2010 were selected as validation set. The characteristics of those patients are listed in Table 1. The median follow-up is 39.4 months in training set and 28.6 months in validation set.

**Table 1:** Characteristic of training set and validation set

	Training set(n=817)		Validation set(n=241)		p
	NO. of patients	%	NO. of patients	%	
Age (years)					0.450
Median	57.7±11.9		58.7±11.4		
Range	19 to 89		21 to 81		
Sex					0.295
Male	547	67.0	170	70.5	
Female	270	33.0	71	29.5	
Tumor size (cm)					0.170
Mean±SD	4.6±2.7		4.3±2.4		
Range	0.2 to 21.0		0.8 to 14.0		
Tumor location					0.555
Upper	342	41.9	93	38.6	
Middle	160	19.6	46	19.1	
Lower	315	38.6	102	42.3	
Pathology type					0.008
Differentiated	289	35.4	108	44.8	
Undifferentiated	528	64.6	133	55.2	
Depth of invasion					0.001
Mucosa or submucosa	107	13.1	35	14.5	
Proper muscle	82	10.0	32	13.3	
Subserosa	163	20.0	104	43.2	
Serosa	363	44.4	60	24.9	
Adjacent invasion	102	12.5	10	4.1	
Positive LN (Mean±SD)	6.2±8.5		5.7±7.7		0.380
Total LN (Mean±SD)	25.0±11.7		24.6±11.1		0.692
mLNR (Mean±SD)	0.2±0.3		0.2±0.3		0.138
AJCC 7th Stage					0.002
IA	83	10.2	29	12.0	
IB	61	7.5	22	9.1	
IIA	57	7.0	32	13.3	
IIB	134	16.4	36	14.9	
IIIA	88	10.8	34	14.1	
IIIB	167	20.4	48	19.9	
IIIC	227	27.8	40	16.6	
CEA (ug/ml)	11.3±52.4		12.5±57.6		0.676
Total Protein (g/l)	67.2±6.7		68.9±6.2		0.155
Albumin (g/l)	41.2±4.3		41.5±3.9		0.226
Globin (g/l)	26.0±4.4		27.3±4.2		0.448
C-reactive protein (mg/l)	8.2±19.9		9.3±31.0		0.337
CAR(mg/g)					0.245
≤0.05	418	51.2	124	51.5	
0.05-0.18	212	25.9	75	31.1	
≥0.18	187	22.9	42	17.4	
WBC (×10^9^/l)	6.6±1.8		6.5±1.6		0.066
Hemoglobin (g/l)	123.0±24.4		129.1±24.3		0.105
Neutrophil (×10^9^/l)	4.0±1.5		4.1±1.4		0.083
Lymphocyte (×10^9^/l)	1.9±0.6		1.8±0.6		0.063
Palate (×10^9^/l)	252.9±97.2		244.1±79.5		0.074
NLR					0.936
≤1.30	136	16.6	19	7.9	
1.30-3.71	589	72.1	190	78.8	
≥3.71	92	11.3	32	13.3	

Abbreviation: LN, lymph node; mLNR, metastatic lymph node ratio; AJCC, American joint Committee on Cancer; GB, globin; CAR, C-reactive protein to albumin ratio; NLR, neutrophil to lymphocyte ratio.

### Independent risk factors in the training set

In the current study, the best cutoff points for C-reactive protein to albumin ratio (CAR) were 0.05 and 0.18 (Supplementary Figure S1). And the best cutoff points for Neutrophil to lymphocyte ratio (NLR) were 1.30 and 3.71 (Supplementary Figure S2). Univariate analysis showed that the age at diagnosis, location, pathological type, depth of invasion, mLNS, mLNR, AJCC 7th stage, NLR, ALB, CRP, CEA and CAR were associated with OS. Multivariate analysis with Cox PH regression showed that age at diagnosis, location, pathological type, depth of invasion, mLNS/mLNR, NLR and CAR were independent risks for overall survival (Table 2). Based on the multivariate analysis, six models were constructed: AJCC staging system; Nomogram A based on AJCC staging system and SIRMs; Nomogram B based on mLNS; Nomogram C based on mLNR; Nomogram D based on mLNS and SIRMs; Nomogram E based on mLNR and SIRMs.

**Table 2:** Multivariate Analysis of the training set

	Hazard ratio	95% CI	*p*
Age	1.02	1.01 to 1.03	<0.001
Location			<0.001
Lower	ref		
Middle	1.62	1.17 to 2.24	
Upper	1.81	1.38 to 2.36	
Pathological type	0.64	0.50 to 0.81	<0.001
Depth of invasion			<0.001
Mucosa or submucosa	ref		
Proper muscle	2.14	0.88 to 5.23	
Subserosa	2.88	1.28 to 6.42	
Serosa	3.97	1.83 to 8.64	
Adjacent invasion	5.11	2.28 to 11.45	
mLNR	7.99	5.47 to 11.67	<0.001
CAR			<0.001
≤0.05	ref		
0.05-0.18	1.32	1.00 to 1.75	
≥0.18	2.27	1.73 to 2.97	
NLR			0.019
≤1.30	ref		
1.30-3.71	1.41	0.96 to 2.06	
≥3.71	1.91	1.21 to 3.03	

Abbreviation: mLNR, metastatic lymph node ratio; NLR, neutrophil to lymphocyte ratio; CAR, C-reactive protein to albumin ratio.

### Development and validated the nomogram for gastric cancer

**Figure 1:**
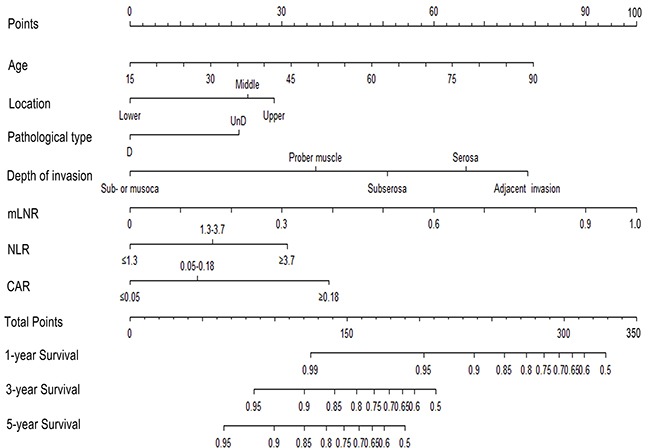
Nomogram predicting 1-year, 3-year and 5-year OS for RGC patients after D2 resection The nomogram is used by adding up the points identified on the points scale for each variable. According to the sum of these points projected on the bottom scales, the nomogram can provide the likehood of 1-year, 3-year and 5-year OS for an individual patient. Abbreviation: mLNR, metastatic lymph node ratio; NLR, neutrophil to lymphocyte ratio; CAR, C-reactive protein to Albumin ratio; OS, overall survival; RGC, resectable gastric cancer.

The risk factors which were statistically significant in multivariate analysis were incorporated intothe prognostic nomogram (Figure 1). When the nomogram including depth of invasion, mLNS, age, location, pathological type and SIRMs, the C-index for predicting OS was 0.77, which were significantly lower than that of the nomogram including depth of invasion, mLNR, age, location, pathological type and SIRMs (0.80; p =0.005). It suggests that the discrimination power of mLNR is better than mLNS in this nomogram. Additionally, according to the total score identified on the points scale, the current nomogram clearly assigned the probability of 1-year, 3-year and 5-year OS.

### Validation of predictive accuracy for OS of the nomogram

**Figure 2:**
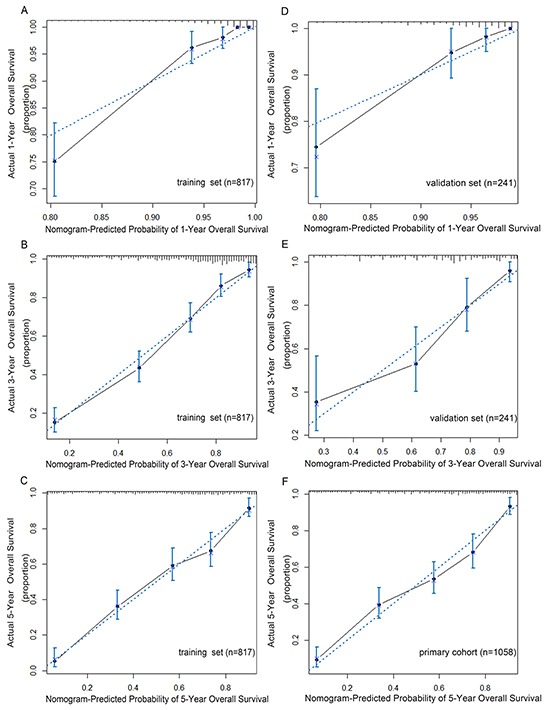
The calibration curve for predicting patients overall survival at 1-year **A.** 3-year **B.** and 5-year **C.** in the training set and predicting overall survival at 1-year **D.** and 3-year **E.** in the validation set, 5-year **F.** in the primary cohort. The X-axis represents the nomogram-predicted survival, and the actual survival is plotted on the Y-axis. The dotted line represents the ideal correlationship between predicted and actual survival.

**Figure 3:**
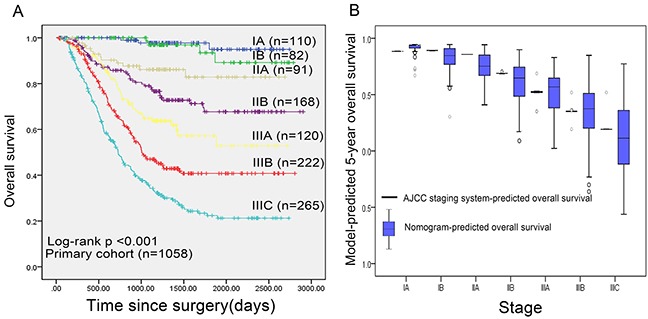
**A.** Overall survival by AJCC 7th staging system in primary cohort; **B.** Distribution of nomogram-predicted 5-year overall survival within each AJCC 7th stage grouping.

In the validation set, the C-index of the nomogram included mLNR for predicting OS was 0.79 (95% CIs 0.74-0.84; p<0.001), higher than that of AJCC 7th staging system(C-index=0.70; 95% CIs 0.64-0.76; p<0.001). Furthermore, as shown in Figure 2, the probability of 1-year, 3-year and 5-year survival in this nomogram corresponded closely between prediction and observation. In addition, Figure 3A shows the Kaplan-Meier survival curve of the primary cohort categorized by the AJCC 7th staging system, with no good discrimination between stage I and stage II patients. However, by the proposed nomogram, a wider range of predicted survival than AJCC-TNM staging system could be clearly identified within each TNM categories (Figure 3B).

### Comparison of predictive accuracy for OS in different nomogram models

**Figure 4:**
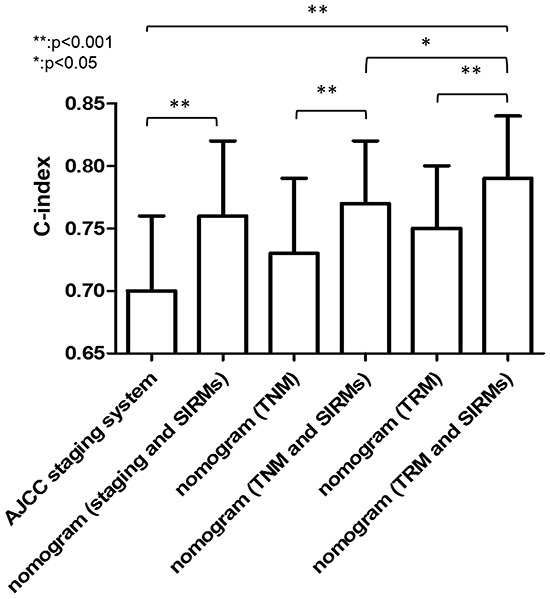
The C-index for OS in different nomogram models Nomogram A including depth of invasion, mLNS, age, location and pathological type; Nomogram B including depth of invasion, mLNR, age, location and pathological type; Nomogram C including depth of invasion, mLNS, age, location, pathological type and SIRMs. Nomogram (proposed) including depth of invasion, mLNR, age, location, pathological type and SIRMs. Abbreviation: mLNS, metastatic lymph node stage; mLNR, metastatic lymph node ratio.

As shown in Figure 4, our nomogram displayed optimal outcome-predicting accuracy in the validation set. The C-index of the proposed Nomogram was 0.79, which was higher than the AJCC 7th staging system (0.70), the potential Nomogram A (0.76, P<0.001), the potential Nomogram B (0.73, P<0.001), the potential Nomogram C (0.75, P<0.001) and the potential Nomogram D (0.77, P=0.005). These results indicated that the proposed nomogram was a useful predictor in predicting both short- and long-term survival of patients with RGC after D2 resection.

## DISCUSSION

In the present study, we established a nomogram based on all the selected independent risk factors for RGC patients after D2 gastrectomy. Especially, inflammatory response markers were included in nomogram for the first time and showed a much more precise prediction for the prognosis of RGC.

When we constructed a nomogram that only included age at diagnosis, location, depth of invasion, mLNS, and pathological type, the C-index for OS prediction was 0.73, higher than that of the AJCC 7th staging system (0.73 vs 0.70, respectively). The result is consistent with the previous studies. For example, Han et al developed a nomogram to predict the probability of 2-year and 5-year survival in 2012 [25]. Hirabayashi et al also constructed and validated a nomogram for overall survival in serosa-negative, locally advanced gastric cancer [26]. Both of the nomograms show more accurate prognosis prediction than the TNM staging system.

Recently, mLNR has been proved to be superior to the number of positive lymph node in the accuracy of prognosis prediction [4, 6, 27, 28]. In this study, mLNR was also testified as an independent risk factor for gastric cancer. Interestingly, when mLNS was replaced by mLNR in training set, the C-index raised from 0.73 to 0.75, which was consistent with previous study [29]. Furthermore, when we added routine detected SIRMs into the nomogram, C-index of our nomogram raised to 0.79. Therefore, the proposed nomogram based on mLNR and other clinical pathology characteristics and SIRMs could make a much more precise prediction than other models for survival of RGC patients after D2 gastrectomy.

Indeed, the pathogenesis of tumor is closely linked to inflammatory. By promoting cell proliferation and angiogenesis, the inflammatory response can stimulate carcinogenesis [30]. Currently, we collect white blood cell count, neutrophil count, lymphocyte count, platelet count, total protein, albumin (ALB), globulin and C-reactive protein (CRP) in the training set. In contrast to other inflammation-related cytokines, the included SIRMs in this study is readily available and relatively cheap. All of them are evaluated in daily clinical routine worldwide [19].

During the past years, many studies have demonstrated the prognostic value of these routine detected SIRMs in gastric cancer patients. For example, Nozoe et al investigated 232 cases and pointed that Glasgow prognostic score (GPS), an inflammation-based prognostic score that combines CRP and ALB, can predict the prognosis of patients with operable gastric cancer. [20] Jung et al detected the significance of the NLR in late stage gastric cancer following resection. They suggested that the elevated preoperative NLR could predict the survival of patients and be utilized as a reliable prognostic indicator for risk stratification [21].

Some possible mechanisms could explain the relationship between the SIRMs and prognosis in RGC patients. First, these SIRMs like the high CRP level, could reflect progressive nutritional and functional decline of gastric cancer patients [31]. Second, these indexes are associated with the inherent immune system, such as macrophage function [32]. In addition, inflammatory response can directly promote angiogenesis and tumor metastasis [14].

There are several potential limitations in our study. Firstly, because of the insufficient of samples and observations, several known variables may not be included in the nomogram models. Secondly, the current nomogram was developed based on data obtained from China. Different from the Japan and Korea, most gastric cancer patients in China are often treated at advanced stage. Whether this nomogram is applicable to other regions is still uncertain.

To the best of our knowledge, this study is the first attempt to develop a prognostic nomogram which combines clinicopathology characteristics and routine detected SIRMs. Compared with previous prognostic models and the AJCC 7th staging system, the current nomogram represents the optimum prognostic discrimination and a better predictive accuracy for survival. It can be used to calculate individualized survival prediction and provide better treatment allocation after D2 gastrectomy. However, further validation whether this nomogram is applicable to all the RGC patients is required.

## MATERIALS AND METHODS

### Patients

From January 1, 2005 to December 31, 2010, among gastric cancer patients after D2 gastrectomy at the Department of Gastric and Pancreatic Surgery at the Sun Yat-sen University Cancer Center, we collected the data of RGC patients who met the following inclusion criteria: no history of receiving anti-cancer therapy before surgery; no history of chronic infectious diseases (e.g. hepatitis, tuberculosis) or inflammatory diseases; no history of liver and kidney dysfunctions; no history of other malignancies; complete resection of cancer with D2 lymphadenectomy. Ethical approval was obtained before surgery.

The data of patients' clinicpathological characteristics (location, size, histology, depth of invasion, mLNS, mLNR) and the routine pretreatment blood SIRMs (white blood cell count, neutrophil count, lymphocyte count, platelet count, total protein, ALB, globulin, CRP were collected. The tumor location was categorized as upper, middle and lower by the center of the lesion. Histology was categorized by differentiated type (papillary adenocarcinoma, well-differentiated tubular adenocarcinoma, and moderately differentiated tubular adenocarcinoma) and undifferentiated type (poorly differentiated tubular adenocarcinoma, signet ring cell carcinoma, and mucinous adenocarcinoma) [25]. The depth of invasion was categorized by mucosa or submucosa, proper muscle, subs-serosa, serosa and adjacent invasion. The pathological tumor stage (IA, IB, IIA, IIB, IIIA, IIIB or IIIC) and mLNS (0, 1~2, 3~6, 7~15 or >16) were categorized according to the AJCC 7th TNM staging system. [3] The mLNR was calculated as the number of positive lymph nodes divided by the number of examined lymph nodes. NLR was defined as neutrophil count divided by lymphocyte count. The CAR was defined as CRP divided by ALB. Follow-up duration was measured as the time from the date of surgery to the last follow-up. OS was defined as the time form surgery to death due to any cause or the last follow-up. The survival status was recorded according to the latest follow-up.

### Follow up

Postoperative follow-up included clinical and laboratory examinations every 3 months for the first 2 years, every 6 months from the 3rd to 5th year, annually after the gastrectomy or until the patient died.

### Statistics analysis

Based on clinical findings, categorical variables were grouped before modeling. The best cutoff points for continuous variables were performed by X-tile (http://www.tissuearray.org/rimmlab/). The continuous variables were divided into three groups. Associations between each group can be calculated by various standard statistical tests, including the log-rank test for survival and means tests for associations between other marker data. The X-tile can provide the optimal division of the data by P values obtained from a lookup table [33].

Independent risk factors were identified by the forward method. OS estimation and survival curves were performed by the Kaplan-Meier method and valided by the log-rank test.

Nomogram was established based on the training set data, and Cox PH regression was used for screening independent risk factors. On the basis of all the independent prognostic factors, a nomogram was constructed by using the package of *rms* in R software version 3.1.3 (http://www.r-project.org/) for predicting 1-year, 3-year, 5-year overall survival. We used the method of bootstraps with 1000 resample for these activities. Harrell's C-index was used in the nomogram for evaluating the discrimination [34]. It can estimate the probability of concordance between the observed and predicted OS. The higher the C-index, the more precise was the survival prediction.

The validation was carried out by using the validation set. Discrimination between the proposed nomogram and AJCC 7th staging system was performed with the *roccp.cens* package in R soft. According to the nomogram-predicted probabilities, calibration were carried out by grouping all the training set patients, validation set and primary cohort, then the mean of the groups were compared with observed Kaplan-Meier OS estimation. P value <0.05 was considered to be statistically significant. All analyses were performed by the software statistical package for social sciences version 19.0 (SPSS, Chicago, IL) and the R software version 3.13 (http://www.r-project.org/).

## SUPPLEMENTARY FIGURES


