# Supplementary material for: Nomogram based on systemic inflammatory response markers predicting the survival of patients with resectable gastric cancer after D2 gastrectomy
Source: Oncotarget. 2016 Apr 18;7(25):37556–65. doi: 10.18632/oncotarget.8788 (PMC5122331; doi:10.18632/oncotarget.8788)
Supplement: Supplementary file 1 [file oncotarget-07-37556-s001.pdf]

Nomogram based on systemic inflammatory response markers predicting the survival of patients with resectable gastric cancer after D2 gastrectomy

SUPPLEMENTARY FIGURES

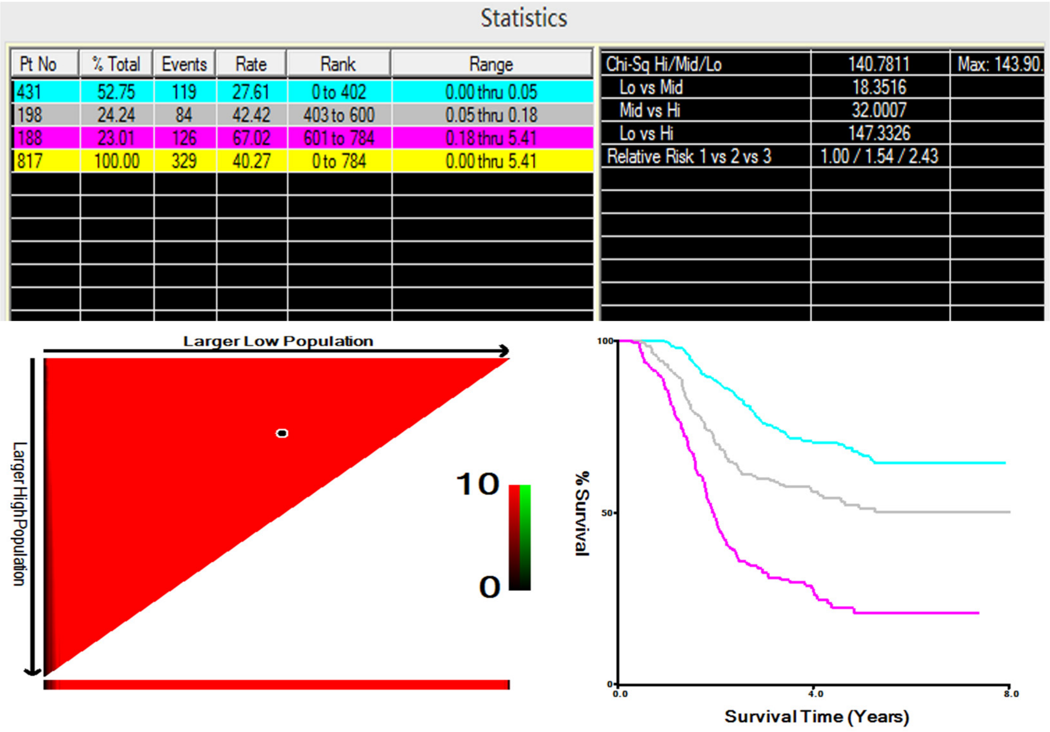

Supplementary Figure S1: X-title analysis of survival data from the training set identified the best cutoff points for C-reactive protein to albumin ratio.

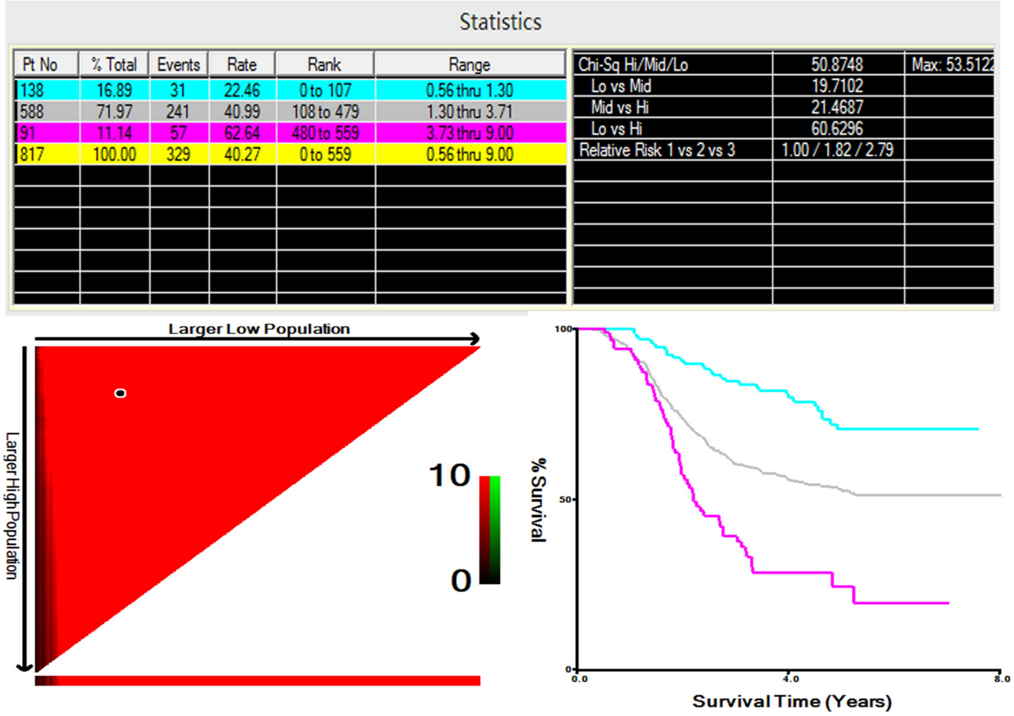

Supplementary Figure S2: X-title analysis of survival data from the training set identified the best cutoff points for neutrophil to lymphocyte ratio.
